# Impacts of Aging and Relative Humidity on Properties of Biomass Burning Smoke Particles

**DOI:** 10.1021/acsestair.4c00224

**Published:** 2024-12-06

**Authors:** Sofie K. Schwink, Liora E. Mael, Thomas H. Dunnington, Maximilian J. Schmid, Jonathan M. Silberstein, Andrew Heck, Nicholas Gotlib, Michael P. Hannigan, Marina E. Vance

**Affiliations:** † Environmental Engineering Program, University of Colorado Boulder, 1111 Engineering Drive, Boulder, Colorado 80309-0428, United States; ‡ Department of Mechanical Engineering, University of Colorado Boulder, 1111 Engineering Drive, Boulder, Colorado 80309-0427, United States; § Department of Aerospace Engineering, University of Colorado Boulder, 429 UCB, 3775 Discovery Drive, Boulder, Colorado 80303, United States

**Keywords:** aerosol, wildfire, organic carbon, liquid water content, hygroscopicity, effective density

## Abstract

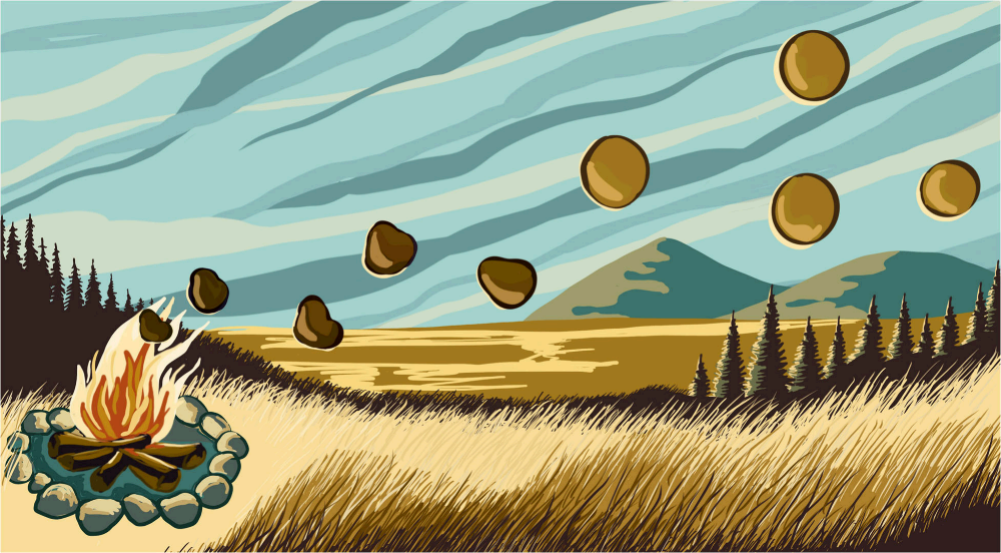

Quantifying changes in the properties of smoke aerosols under varying conditions is important for understanding the health and environmental impacts of exposure to smoke. Smoke composition, aerosol liquid water content, effective density (ρ_eff_), and other properties can change significantly as smoke travels through areas under different ambient conditions and over time. During this study, we measured changes in smoke composition and physical properties due to oxidative aging and exposure to humidity. We found that smoke aging led to SOA formation and increases in ratios of organic carbon to elemental carbon. Aerosol liquid water content increased with increasing relative humidity (RH), and aged smoke took up more water than fresh smoke at all humidity levels, likely due to a combination of changes in aerosol surface polarity at low and medium RH and increases in surface area with aging at high RH. Growth factors ranged from 1.06 ± 0.08 for fresh smoke at low RH to 1.32 ± 0.08 for aged smoke at high RH. Oxidative aging and exposure to humidity led to increases in ρ_eff_. For 100 nm particles, ρ_eff_ ranged from ∼1.2 for fresh smoke at low RH to ∼1.6 for aged smoke at high RH. Results from these experiments suggest that exposure to humidity leads to smoke restructuring and compaction and/or changes in surface chemistry.

## Abstract 2

Oxidative aging and exposure to humidity lead to changes in the physical properties of smoke particles. We measured changes in composition, aerosol liquid water content, and effective density for fresh and aged smoke at three humidity levels.

## 1 Introduction

Emissions from wildfires make up a significant portion of ambient particulate matter (PM) and PM precursors around the world.^1^ Climate change and human impacts on the environment are leading to increased frequency and severity of wildfires, and these impacts are likely to become more intense in the future.^2,3^ Smoke emissions from wildfires can quickly travel long distances and experience different atmospheric conditions over time, altering their physical and chemical properties^4^ and changing their environmental impacts and health effects of exposure. The health effects of exposure to smoke aerosol are more significant than those of exposure to other fine PM.^5^ However, the toxicity of biomass burning (BB) secondary organic aerosol (SOA) has been shown to decrease with aerosol aging,^6^ while oxidative stress caused by reactive oxygen species in smoke is enhanced by aging.^7,8^ Changes in other aerosol characteristics may also have important effects on toxicity.

As smoke travels through the atmosphere, particles and gas-phase compounds are exposed to ultraviolet radiation from the sun and oxidants such as ozone, which promote aging of primary aerosol and formation of SOA.^9^ Aging causes changes in physical and chemical properties of smoke particles, including the relative concentrations of brown carbon (BrC) and black carbon (BC), or soot.^10^ Smoke composition and BrC/BC ratios are largely dependent on combustion conditions, with higher efficiency combustion leading to more BC formation and lower efficiency combustion and oxidative aging leading to proportionally more BrC formation.^11^ Oxidative aging can also change the surface area and polarity of smoke particles, which leads to changes in interactions with water and other compounds in the atmosphere.^12^

Relative humidity (RH) varies dramatically temporally and over horizontal and vertical distances in the atmosphere.^13^ Exposure to different RH levels can lead to changes in particle size, properties, and lifetime.^14^ Variation in ambient RH causes changes in aerosol liquid water content (ALWC), which affects size distributions, toxicity, and other aerosol properties.^15^ ALWC can be measured experimentally or quantified by using models. One popular method for characterizing ALWC is κ-Köhler theory, which relates the hygroscopicity parameter kappa (κ), a quantitative measure of water uptake, to the dry volume of a particle and is often applied to cloud condensation nuclei.^16^ Experimental approaches vary but often rely on measurements of dry and wet particle size distributions. These can use a hygroscopic-tandem differential mobility analyzer (H-TDMA) or a similar system,^17^ or two scanning mobility particle sizers (SMPSs) in parallel, where one SMPS samples a dehumidified stream and another samples unconditioned particles. Interactions between atmospheric liquid water and aerosol particles affect the particle fate and transport, radiative forcing, and hydrological cycling. Aerosols can serve as cloud condensation nuclei, as they rise through the atmosphere and take up water. The presence of water in particles can lead to aqueous-phase chemistry, which affects aerosol composition and physical properties,^18^ including their effective density (ρ_eff_).^19^

Knowing aerosol ρ_eff_ is important for understanding particle dynamics in the atmosphere, in measurement instruments, and in the human respiratory system.^20^ It is also important for converting particle number concentrations measured by an instrument, such as an SMPS, to mass concentrations. Typically, unit density is used to make this conversion, which generally leads to underestimations of total mass concentrations.^21^ Size and density are two of the most significant factors that determine how particles deposit in the atmosphere and the human respiratory system,^22^ so it is crucial that we gain a better understanding of aerosol density in laboratory and real-world environments.

Many studies use an assumed ρ_eff_ for smoke between 1.0 and 1.8 g cm^–3^, with ∼1.4–1.5 g cm^–3^ being a typical assumption, to convert from number concentrations to mass concentrations.^23−28^ However, smoke ρ_eff_ can vary due to changes in combustion conditions, level of oxidative aging, exposure to humidity, and other factors.^29−32^ Atmospheric models often use an assumed aerosol density when modeling aerosol sedimentation velocity, aggregation, and transport in the atmosphere.^33^ Similarly, low-cost sensors are calibrated by using an assumed particle density. Differences in density influence light scattering and absorption in light-based aerosol measurement devices like low-cost sensors, so there is a need to better characterize how changes in density affect these measurements.^34^ Moreover, particle density can provide insight into particle composition, mixing state, and aging.^35^

Although using ρ_eff_ instead of material density to convert from number to mass concentrations may lead to underestimation of mass concentrations, ρ_eff_ is a useful metric in determining how particle morphology changes under varying conditions. ρ_eff_ is connected to morphological and chemical properties of aerosols and helps to link material density to the dynamic shape factor.^36^ It is also correlated with aerosol refractive index and affects aerosol optical properties.^37^

Smoke ρ_eff_ has been measured for a range of environmental and combustion conditions, but there are still major inconsistencies in density assumptions made to convert smoke number concentrations to mass concentrations.^27^ More insight into how smoke ρ_eff_ changes with exposure to RH, oxidative aging, use of different fuels, and different combustion conditions is necessary to better understand smoke dynamics in the atmosphere and the potential health impacts of exposure.

The objective of this work was to determine how oxidative aging and exposure to humidity affect the physical and chemical properties of smoke aerosols in controlled chamber experiments. We assessed changes in smoke composition due to oxidative aging using gas chromatography–mass spectrometry (GC-MS) and measurements of elemental carbon (EC) and organic carbon (OC) concentrations in fresh and aged smoke. We investigated smoke optical properties by performing aethalometry measurements of BC and BrC concentrations. Then, we quantified ALWC for fresh and aged smoke at three RH levels. Finally, we measured ρ_eff_ of the nonwater components of fresh and aged smoke that had been exposed to different RH levels. Understanding how the properties of smoke change under different atmospheric conditions can help inform us about changing impacts on human health, the environment, and climate.

Other groups have investigated how aging affects the chemical composition of the BB particles. Reid et al. found that condensation of organic species onto primary BB particles can increase the total particle mass by up to 40%, and the total fraction of semivolatile compounds in smoke increases substantially during aging.^38^ Cappa et al. measured changes in aerosol absorptivity and other properties following photochemical aging for a variety of fuel types, finding that BrC absorptivity decreases after aging due to SOA production.^10^ Martin et al. observed a gradual increase in hygroscopicity parameter values for smoke aerosols after turning on a UV light to age primary particles.^39^ A variety of studies have been conducted investigating changes in these properties individually, but few studies have measured changes in a broad variety of chemical, optical, and physical properties like we have done for this work.

## 2 Materials and Methods

### 2.1 Experimental Setup

**Figure 1:**
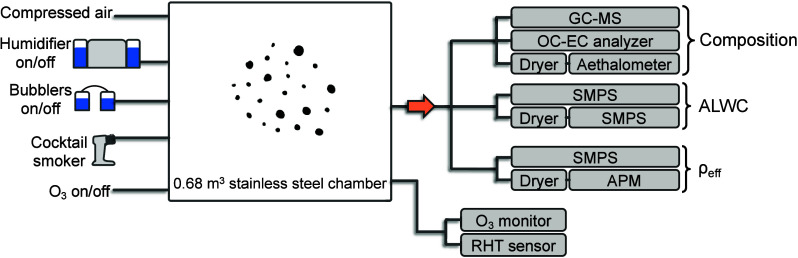
Schematic of the experimental chamber setup. A combination of dry air, air that passed through an evaporative humidifier located inside a separate chamber, and air that passed through bubbler bottles was used to create the RH levels of interest.

For all experiments, smoke was generated by burning ∼5 mg of ponderosa pine wood chips in a cocktail smoker (the Smoking Gun, BSM600SILUSC, Breville, Sydney, Australia), which we also used in the recent Chemical Assessments of Surfaces and Air (CASA) indoor field study.^32^ Smoke was injected through 0.75 m of 0.4 in. (10 mm) i.d. silicon tubing into a 0.68 m^3^ stainless-steel chamber (Figure 1). The chamber was held at one of three RH levels: “low” at 0–10%, “medium” at 50–60%, or “high” at 80–90%. The chamber either contained no ozone for fresh smoke experiments or 2.9 ± 0.6 ppm of ozone to rapidly simulate smoke aging. RH and temperature were measured downstream of the chamber during each experiment using an aerosol humidity and temperature sensor (RHT 3000, TSI, Shoreview, MN).

To achieve low RH, we passed ∼8 lpm of clean, dry air through the chamber. For medium RH, we passed ∼5 lpm through a well-sealed plastic tub containing an evaporative humidifier plus ∼3 lpm of makeup dry air into the chamber. For high RH, we passed ∼5 lpm through the humidifier tub and ∼3 lpm through two 0.5 L bubbler bottles containing purified water for at least ∼5 h, usually overnight. Different online instruments and samplers for offline measurements (described below) sampled from the chamber, and excess airflow was allowed to exit passively into a fume hood through 7/8 in. (22 mm) i.d. silicon tubing. For simulated aged smoke experiments, a UV ozone generator (M610, Jelight, Irvine, CA) was used to generate a high concentration of ozone (2.9 ± 0.6 ppm) in the chamber. The chamber was kept dark during each experiment, and we did not add OH radicals; therefore, the primary reactions occurred between smoke and ozone. At least two trials were performed for each experimental condition. Aerosol composition and EC/OC, ALWC, and ρ_eff_ were not measured concurrently, but rather in separate experiments. Aerosol size distributions, ozone concentrations, and environmental parameters were measured in every trial. Average peaks in the size distribution for all experimental conditions are shown in Table S1.

Data collection occurred over 75 min following each smoke injection for ALWC and ρ_eff_ experiments. We performed one extended experiment with a total measurement time of 150 min after smoke injection to ensure results did not change and significant reactions were not occurring with increased residence time in the chamber. After each experiment, the chamber was flushed with filtered, compressed air for at least 12 h, usually overnight, to reduce particle concentration to near zero before beginning the next experiment.

Because fuel moisture content can affect emissions from biomass burning, we assessed the moisture content of the ponderosa pine wood chips by baking 1 g of them in an oven at 120 °C for 8 h. The wood chips were weighed before and after baking, and their difference in mass was used to determine moisture content, which was 5.4%.

### 2.2 Aerosol Composition and Optical Property Measurements

To assess how the chemical properties of smoke change with aging, we performed composition experiments in a low RH experimental setting. Samples were passed through quartz fiber filters (QFF) for 100 min after smoke injection to ensure there was sufficient time for particles to deposit during each experiment. PM_2.5_ was collected using an emission pod (Hannigan Lab, Boulder, CO) with flow rates calibrated to 2 lpm. The inlet for the PM sampling device was connected to the chamber via 0.2 m of 1/4 in. (6 mm) i.d. conductive silicon tubing. PM_2.5_ was collected on 25 mm o.d. QFFs. Before collection, QFFs were conditioned at 500 °C for 12 h to remove any preexisting organics.

We measured particulate EC and OC concentrations from QFFs with an OC-EC analyzer (Lab OC-EC Aerosol Analyzer, Sunset Laboratory, Tigard, OR). EC and OC constituents as well as total EC and OC concentrations were determined for each of the QFFs using the NIOSH 5040 protocol and the thermal-optical-transmittance method. OC and EC loadings from a pretreated blank were subtracted from all experimental measurements. Uncertainties were calculated using the root sum of squares method.

We measured black carbon (BC) and brown carbon (BrC) concentrations during low RH experiments using a 5-wavelength aethalometer (microAeth MA200, Aethlabs, San Francisco, CA). The aethalometer was connected to the chamber outlet through 0.15 m of 1/4 in. (6 mm) i.d. silicon tubing and a 0.5 m diffusion dryer with silica beads, which created very low RH (<1%). The aethalometer collected data in dual spot mode for ∼15 min of background scans and then for ∼20 min after smoke injection. The first few minutes of data after smoke injection and data points immediately following each tape spot switch were removed from our analysis due to brief instability in measurements after spot switches.

We performed organic compound measurements using an Agilent Gas Chromatograph Mass Spectrometer (6090N GC, 5975 MS, Santa Clara, CA).^40,41^ Following methods outlined in Dutton and Xie,^42,43^ organic chemical analysis was run via solvent extraction of QFFs with dichloromethane followed by GC-MS. Additional details regarding the extraction and analysis methodology can be found in Silberstein et al.^44^ Filters from the two experimental trials with the greatest differences in OC/EC and BrC/BC ratios between fresh and aged smoke were selected for organics analysis using GC-MS. At the temperatures maintained during these experiments, which were well below the melting points of chemical compounds we detected in smoke samples, we expect that primary particles are mostly solid or semisolid and that SOA is semisolid or glassy.^45^ After hygroscopic growth during medium and high RH experiments, smoke aerosols are expected to be in a mostly semisolid or aqueous state.^46^

A scanning mobility particle sizer (SMPS 3082, TSI), consisting of a long differential mobility analyzer (DMA 3081, TSI) and a butanol-based condensation particle counter (CPC 3750, TSI), was used in parallel with the other instruments to monitor particle concentrations and size distributions in the chamber.

### 2.3 Aerosol Liquid Water Content Measurements

We used two SMPS systems in parallel to measure differences in particle size distributions attributable to the presence of water in fresh and aged smoke at low, medium, and high RH. Air from the chamber was sampled by both SMPS systems simultaneously. One SMPS (3082, TSI), equipped with a long differential mobility analyzer (DMA 3081, TSI) and a butanol-based condensation particle counter (CPC 3750, TSI), sampled aerosol directly from the chamber and was used to determine the “wet” particle volume (*V*_wet_). The other SMPS (3080, TSI), which was operated with a long DMA (3081, TSI) and a water-based CPC (3788, TSI), was used to determine dry particle volume (*V*_dry_) by sampling air through a 0.5 m long silica bead diffusion dryer, which removed water associated with particles. The silica beads in the dryer were dried for 2 h at 135 °C in an oven after each medium and high RH experiment. The RH downstream of the dryer was measured in the range of ∼0.1–0.5%. The SMPS systems were operated in parallel overnight between experiments to allow them to reach equilibrium at each humidity level before the next experiment. Aerosol liquid water content (ALWC) as a fractional portion of total wet aerosol volume was determined using eq 1:
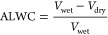
1

The volume growth factor (GF_vol_), which gives information on how particle volume changes with exposure to humidity, was calculated using eq 2(17)
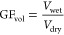
2and the hygroscopicity parameter, kappa (κ), was determined from the volume of water in particles (*V*_water_), *V*_dry_, and the activity of water (*a*_w_) in the chamber using eq 3:^16^
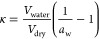
3

The SMPS systems were intercompared at the end of each experiment without the diffusion dryer to determine size-resolved correction factors, which were applied to data from the SMPS 3080 for agreement with the SMPS 3082. Correction factors were created and applied to each experiment individually and generally fell in the range of ∼0.6–1.1.

### 2.4 Effective Density Measurements

We used an aerosol particle mass monitor (APM 3602, Kanomax, Andover, NJ) with a long DMA (3081, TSI) upstream and a water-based CPC (3788, TSI) downstream to measure the mass of 50, 70, 100, 150, 230, and 340 nm smoke particles. A Gaussian curve was fitted to the ρ_eff_ results for each size bin, and peaks were found to determine the most representative ρ_eff_ value for each particle size. We took APM measurements of smoke particles that were exposed to low (0–10%), medium (50–60%), and high (80–90%) RH in the chamber. Most samples were dried using a 0.5 m diffusion dryer before APM measurements to assess changes in ρ_eff_ of the core of the aerosol associated with structural changes following exposure to water and subsequent loss of this water. We performed additional APM measurements at low and high RH without the diffusion dryer to determine the ρ_eff_ of smoke particles with associated water. During these experiments, we used an SMPS (3082) in parallel to monitor particle size distributions and concentrations.

Determining the material density of fractal, nonspherical particles generally requires calculation of the dynamic shape factor.^47^ This calculation involves combining knowledge of electrical mobility diameter measured by an instrument such as an SMPS with vacuum aerodynamic diameter measured by an instrument such as an aerosol mass spectrometer.^48^ For these experiments, we were unable to measure the vacuum aerodynamic diameter, so we calculated ρ_eff_ under the assumption that particles were spherical. Because ρ_eff_ is lower than material density, this approach likely leads to an underestimation of the true aerosol density.^49^ The ρ_eff_ was calculated as a ratio of particle mass (*m*_p_) to mobility diameter equivalent volume (*d*_m_) using eq 4:
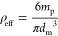
4

## 3 Results and Discussion

### 3.1 SOA Formation during Oxidative Aging

Although the same amount of wood chips was combusted for each experiment (∼5 mg), there were significant differences in the mass concentration of smoke generated for each experimental condition. The aerosol mass concentration was 403 ± 191 μg m^–3^ for fresh smoke and 612 ± 188 μg m^–3^ for aged smoke. The average particle diameter for the first three SMPS scans after smoke injection was smaller for fresh smoke than aged smoke (*D*_fresh_ = 80 ± 7 nm, *D*_aged_ = 102 ± 7 nm). This increase in particle mass concentration and size was likely due to condensation of SOA onto the primary aerosol. We consistently observed an initial small, secondary peak in size distribution at ∼50–70 nm. For fresh smoke experiments, this peak was lost over time due to coagulation, and for aged smoke it remained throughout each experiment due to formation of new, small particles. Size distribution heat maps showing particle formation and growth for fresh and aged smoke experiments at low RH are shown in Figures S1 and S2, respectively.

Concentrations of EC did not change significantly between the fresh and aged smoke samples, as shown in Table 1. However, OC concentrations increased significantly with aging (Table 1). During aged smoke experiments, ozone in the chamber promoted the formation of SOA via repeated oxidation of volatile organic compounds present in the smoke, resulting in increased OC concentrations. The OC-EC analyzer measured higher concentrations of pyrolyzed carbon, commonly associated with SOA, in aged smoke than in fresh smoke (fresh 41 ± 15 μg m^–3^, aged 59 ± 6 μg m^–3^). This relative enhancement of pyrolyzed carbon between fresh and aged smoke samples (∼1.4–1.5×) was similar to the total OC enhancement, which agrees with the literature.^50,51^ Aerosol mass enhancements due to condensation of SOA onto primary smoke particles have been reported by other groups in the range of ∼50–800%.^52,53^

**Table 1:** Concentrations of Organic and Elemental Carbon (OC and EC), Measured by the OC-EC Analyzer, and Black and Brown Carbon (BC and BrC), Measured by the Aethalometer for Fresh and Aged Smoke Experiments at Low RH^a^

	OC (μgC m^–3^)	BrC (μg m^–3^)	EC (μgC m^–3^)	BC (μg m^–3^)
fresh smoke	193 ± 64	132 ± 54	6 ± 2	9 ± 2
aged smoke	290 ± 20	207 ± 27	8 ± 3	11 ± 3

a Averages shown are ± standard deviation.

Measurements from the aethalometer show trends similar to those from the OC-EC analyzer. BC concentration was similar between fresh and aged smoke experiments and BrC concentration was higher for aged smoke than for fresh smoke (Table 1). The increase in the BC/EC between fresh and aged smoke experiments is not statistically significant. The mass concentration of BrC measured by the aethalometer was ∼30% lower than the OC concentration measured by the OC-EC analyzer. The aethalometer relies on the light absorbance of particles continually collected on a filter to determine concentrations of BrC and BC, whereas the OC-EC analyzer heats the sample and converts all carbon-containing material into methane, which is then measured using a flame ionization detector. Optical measurements taken with an aethalometer have inherently higher detection limits and uncertainty than OC-EC measurements and tend to underestimate brown carbon concentrations, as seen in these results, although there are strong correlations between these measurements.^54,55^

### 3.2 Composition Differences between Fresh and Aged Smoke at Low RH

**Figure 2:**
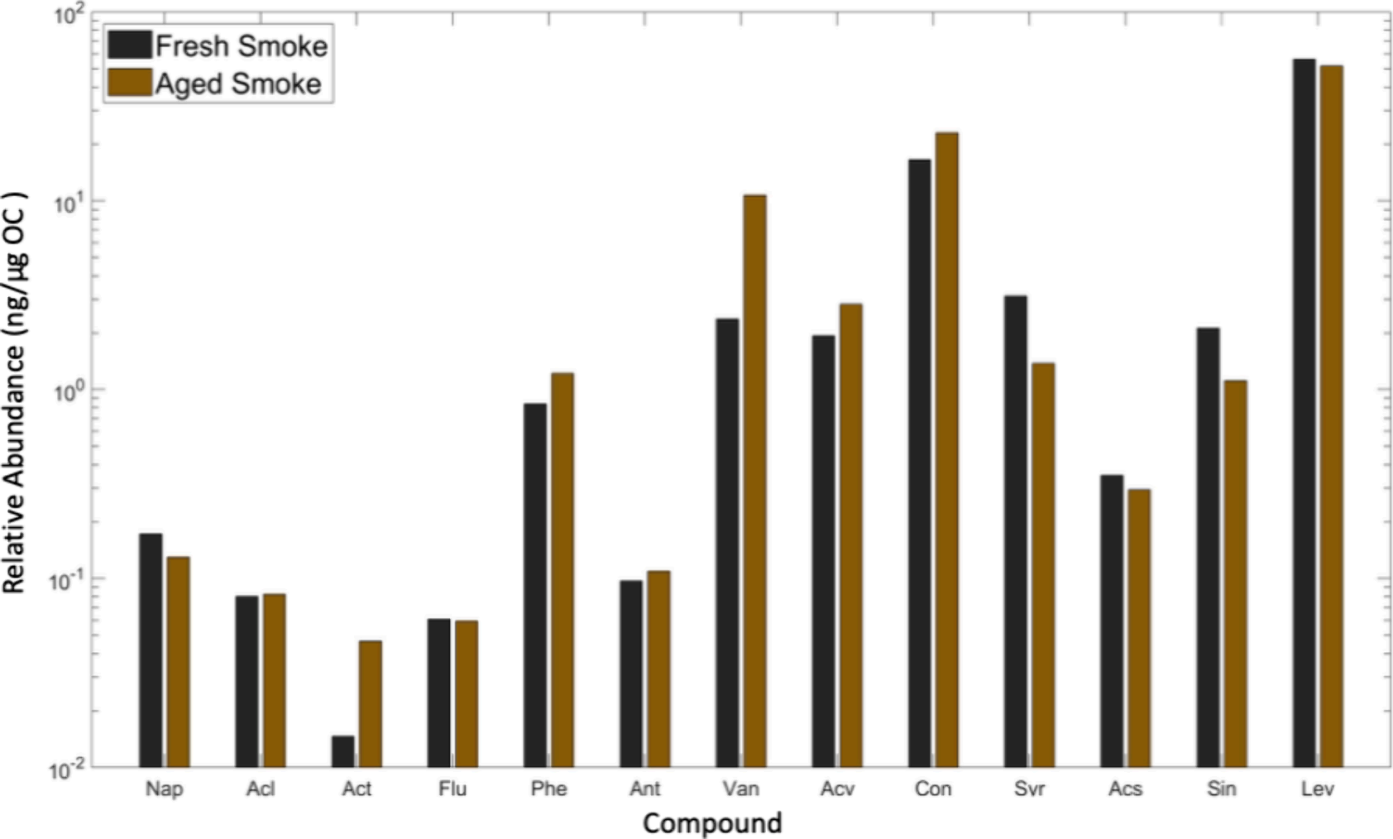
Chemical composition of fresh and aged smoke normalized by the OC concentration. Compounds are grouped by chemical similarity. Key: nap, naphthalene (C_10_H_8_); acl, acenaphthylene (C_12_H_8_); act, acenaphthene (C_12_H_10_); flu, fluorene (C_13_H_10_); phe, phenanthrene (C_14_H_10_); ant, anthracene (C_14_H_10_); van, vanillin (C_8_H_8_O_3_); lev, levoglucosan (C_6_H_10_O_5_); acv, acetovanillone (C_9_H_10_O_3_); con, coniferaldehyde (C_10_H_10_O_3_); syr, syringaldehyde (C_9_H_10_O_4_); acs, acetosyringone (C_10_H_12_O_4_); sin, sinapinaldehyde (C_9_H_8_O); stg. stigmasterol (C_29_H_48_O).

Several small polycyclic aromatic hydrocarbons (PAH) and nonacid carbonyl-containing compounds (i.e., aldehydes and ketones) were identified in fresh and aged smoke particles. These included many organic chemical tracers linked to softwood combustion (Figure 2). In this study, we burned wood chips from ponderosa pine, which is a softwood. Several methoxyphenol compounds, including vanillin and acetovanillone, are found in the cell walls of plants and are more abundant in softwoods than hardwoods.^56^ These compounds are also commonly emitted from the pyrolysis of lignin.^57^ When normalized by the mass of OC, these compounds were found in similar concentrations in fresh and aged smoke, indicating that organic species created during combustion partition from the gas to the solid phase, resulting in secondary aerosol formation via heterogeneous nucleation during aged smoke experiments, as observed in particle size distributions (Figures S1 and S2). Levoglucosan, a common biomass burning marker,^39^ was the dominant species after normalization in both fresh and aged smoke experiments. Lighter PAHs were found in nearly identical concentrations in normalized fresh and aged smoke experiments, again indicating a transition from gas to solid phase as a result of equilibrium dynamics.^58^ A chromatogram from these experiments is shown in Figure S3.

We were able to characterize 44% of the total OC mass by GC-MS for aged smoke experiments and 17% for fresh smoke experiments. The discrepancy between these values is predominantly due to the extremely high concentrations of organic acids in aged smoke samples. Stefenelli et al. measured VOC emissions from flaming and smoldering wood combustion and found that lignin pyrolysis products, polycyclic aromatics, and furans are important SOA precursors emitted from biomass burning.^59^ They found that SOA formation is dominated by the oxidation products of oxygenated aromatics emitted from the pyrolysis of lignin regardless of combustion conditions and oxidative aging. Tkacik et al. measured variable concentrations of alkanes, alkenes, ethyne, aromatics, and terpenes between experiments when burning ponderosa pine.^60^ It is likely that smoke generated in these experiments contained components similar to those measured by Stefenelli et al. and Tkacik et al.

The chemical profile of smoke generated during this study generally aligns with what has been observed from wildfires in the western US, with some differences. Ward et al. measured similar relative concentrations of levoglucosan but smaller relative concentrations of vanillin, acetovanillone, and coniferaldehyde during a study of Montana wildfire smoke than we did from burning Ponderosa pine.^61^ Silberstein et al. observed variation in smoke composition between locations within a small area during a wildfire in Colorado but generally observed trends in concentrations of naphthalene, acenaphthylene, acenaphthene, fluorene, and anthracene similar to what we measured.^44^ Concentrations of phenanthrene were higher during our study than during the measurements of Silberstein et al. Differences in these values are likely due to differences in fuel type, combustion conditions, and ambient environmental conditions. Farmer et al. observed roughly a 1:1 relationship between the relative concentrations of 10 compounds in wildfire smoke from a real fire and smoke generated by burning ponderosa pine in a cocktail smoker like the one used for this study.^32^ Although our bulk aerosol measurements do not provide insight into which specific chemical components of smoke undergo changes that lead to differences in aerosol physical properties, we expect that the changes observed during these experiments are similar to what could occur in the real world.

Vandergrift et al. took measurements of the phase state of carbonaceous particles from wildfire smoke in ambient air in Washington and found that for particles with diameters between 100 and 180 nm, more than half of smoke aerosols were semisolid, approximately a third were liquid, and a small portion were solid.^62^ At night, the proportion of semisolid particles was roughly the same as that during daytime measurements, the proportion of solid components was higher than that during daytime, and the proportion of liquid components was lower than that during daytime. Gregson et al. found that BB organic aerosol from smoldering burns of pine wood has two phases—a hydrophobic phase on particle surfaces and a hydrophilic phase underneath.^63^ They determined that the outer hydrophobic phase was glassy for temperatures of below 230 K and RH values of below 95%. Our experiments were performed at temperatures well below the melting points of the compounds we detected during our GC-MS measurements. The GC-MS was only able to characterize part of the total aerosol mass, but we believe that a significant portion of smoke aerosols was either semisolid or solid during these experiments based on the melting points of these compounds and findings from literature.

### 3.3 Aerosol Liquid Water Content at Different RH Levels and Aging Conditions

**Figure 3:**
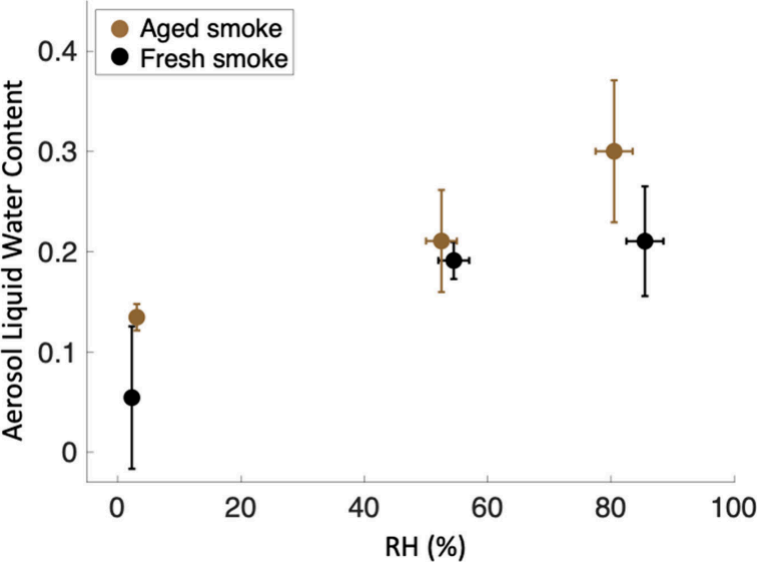
ALWC as a fraction of total aerosol volume for fresh and aged smoke aerosols with increasing RH. The *y*-axis represents the fraction of aerosol volume made up of liquid water. Averages shown ± standard deviation.

Average ALWC as a fraction of total aerosol volume ranged from 5.4 ± 7.1% for fresh smoke at low RH to 30 ± 9% for aged smoke at high RH (Figure 3). ALWC increased with increasing RH, and enhancements were greater for aged smoke than for fresh smoke. All fractional ALWC volumes are shown in Table S2.

The size distributions of particles both before and after drying were in the accumulation mode and had peaks ranging from ∼100 to ∼160 nm. Representative size distributions for fresh and aged smoke experiments with and without a diffusion dryer at low RH are shown in Figure S4. We observed increases in average particle diameter with aging at all humidity levels, with an average increase in diameter of the aerosol core of 19 ± 9 nm measured by the dry SMPS. Information on changes in size distribution measured by the dry and wet SMPS systems can be used to calculate aerosol volume growth factors and the hygroscopicity parameter, κ. Volume growth factors ranged from 1.06 ± 0.08 for fresh smoke at low RH to 1.32 ± 0.08 for aged smoke at high RH (Figure S5) and κ values ranged from 0.10 ± 0.02 for fresh smoke at low RH to 0.28 ± 0.03 for aged smoke at high RH (Figure S6). κ values measured in these experiments were similar to those for biomass burning emissions from other studies. Twohy et al. measured κ between ∼0.05 and 0.15 for ambient biomass burning emissions over the western United States.^64^ Petters et al. measured decreases in κ with increasing particle size during controlled laboratory experiments, with κ values ranging from 0.02 to 0.8 and an average κ for smoke from burning ponderosa pine of 0.06.^65^ Our ALWC measurements were not size resolved. Petters and Kreidenweis found a range of κ between 0.01 and 0.5 for organic species using an atmospheric model.^16^

Aged smoke particles were larger than fresh smoke particles and had more total surface area, thus providing more space for the initial formation of a thin film of water and ultimately more water uptake. To determine if this was the reason for increased water uptake by aged particles or if it was due to another factor, such as changes in surface polarity associated with aging, we normalized ALWC by the dry particle surface area (Table 2). At low and medium RH, normalized water content increased with particle aging. At high RH, normalized water content was similar between fresh and aged smoke.

**Table 2:** ALWC Expressed as a Fraction of Total Aerosol Volume Normalized by Dry Particle Surface Area (μm^–2^)^a^

	Low RH	Medium RH	High RH
Fresh	7.3 ± 1	24 ± 0	43 ± 3
Aged	16 ± 0	43 ± 2	42 ± 4

a Averages shown ± standard deviation.

Increases in surface-area-normalized water content at low and medium RH suggest that aging of the aerosol causes chemical changes to the particle surface that lead to an increase in hygroscopicity, although we were unable to confirm this through GC-MS results. GC-MS is used to determine the bulk aerosol composition and cannot be used to determine what chemistry is happening on particle surfaces. At high RH, the normalized water content was approximately the same between fresh and aged smoke, meaning that each unit of surface area took up the same amount of water on fresh and aged smoke particles. These results suggest that increased water uptake by aged aerosol at high RH was only due to increases in particle surface area following condensation of SOA onto the primary aerosol. Changes in surface chemistry likely did not have a significant impact on aerosol hygroscopicity at high RH.

Bian et al.^15^ performed direct measurements of ALWC in ambient aerosol and saw gradual increases for RH below ∼80%, with an exponential increase above ∼80% RH, which agrees with κ-Köhler theory. The trend of our gradual increases in ALWC aligns with the measurements of Bian et al., although we were not able to achieve sufficiently high RH to observe exponential particle growth.

### 3.4 Aerosol Effective Density (ρ_eff_) at Different RH Levels and Aging Conditions

**Figure 4:**
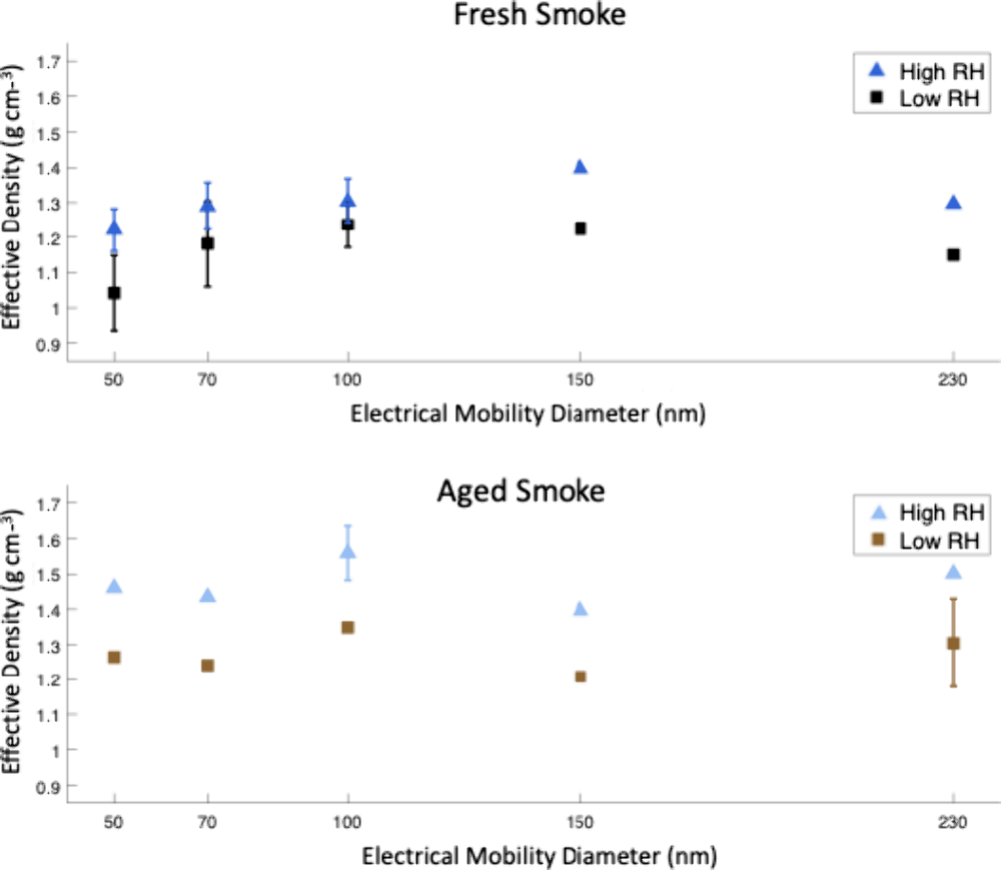
Effective density of fresh smoke (top panel) and aged smoke (bottom panel) that was exposed to either high or low RH and then dried with a diffusion dryer. There were not sufficient concentrations of 340 nm particles to observe a peak in ρ_eff_, so these results have not been reported. Averages shown ± standard deviation.

The ρ_eff_ of smoke aerosols with associated water was similar between fresh and aged smoke in all size bins and for all RH levels, with an average ρ_eff_ for all conditions of 1.16 ± 0.05 g cm^–3^ (Table S3). However, with a diffusion dryer upstream of the APM to remove water associated with particles, the ρ_eff_ of smoke that had been exposed to high RH was higher than the ρ_eff_ of smoke that was exposed to low RH in all size bins, suggesting that exposure to humidity followed by drying leads to compaction of the aerosol structure (Figure 4). For 100 nm particles exposed to high RH and then dried, ρ_eff_ was 1.30 ± 0.07 for fresh smoke and 1.56 ± 0.08 for aged smoke. This result is unexpected because these particles are primarily composed of organic carbon and not soot, as shown by our chemical composition experiments. The ρ_eff_ values of particles exposed to medium RH generally fell between the values for low and high RH experiments but did not follow the same trend (Figure S7). This could be because particles in this humidity range were transitioning between the original and “collapsed” structures. Density values for all experiments with a diffusion dryer are listed in Table S4. Martin et al.^58^ measured similar compaction of soot aerosol following exposure to humidity in particles with a diameter ≥100 nm.

Oxidative aging leads to the condensation of SOA into voids in the primary smoke structure. Because of this, the mass of a spherical particle of a certain volume is greater for aged smoke than for fresh smoke, and thus ρ_eff_ is higher for aged smoke than for fresh.^58^ We measured higher ρ_eff_ values for aged smoke than for fresh smoke for the same humidity conditions in all size bins except 150 nm. ρ_eff_ of 150 nm particles was the same between fresh and aged smoke. This discrepancy could be due to the presence of multiple charges, which has the greatest effect on mass measurements of this particle size bin. Error due to multiple charges can be up to 13% in this size bin if using only one DMA.^66^

Small variations in ρ_eff_ between size bins for each experimental condition were not statically significant, suggesting that changes in particle ρ_eff_ are not size-dependent under these conditions. Other studies have found that ρ_eff_ of smoke aerosols emitted from many types of fuel decreases with increasing particle size if burned under high temperature flaming conditions, in which case combustion efficiency is high and BC dominates emissions.^28^ However, ρ_eff_ is relatively constant for low-efficiency smoldering emissions across all size bins, likely because these emissions have a high BrC component and are not heavily branched.^28,63^ Pokhrel et al.^28^ report ρ_eff_ values for smoldering emissions from six different woods sourced from Africa. These experiments were performed at low RH in the presence of ozone, which is similar to our aged smoke, low RH condition. ρ_eff_ was measured in the range of ∼1.0–1.3 g cm^–3^ and the fuel types with density values closest to ours (from ponderosa pine) were olive and mukusi.

Significant changes in ρ_eff_ are most likely to occur in highly branched soot structures, but it is possible that there was also compaction of BrC/OC during these experiments. There was not enough BC/EC to account for the density increase we measured if BC/EC alone was undergoing structural changes. Ma et al. observed soot particle packing and collapse following exposure to humidity in controlled chamber experiments and found that the amount of packing increased with increasing soot particle size.^67^ They measured volume decreases following exposure to humidity between 14% for 50 nm particles and 44% for 200 nm particles. The average particle packing measured across all size bins during this study was 31 ± 4% for fresh smoke and 15 ± 2% for aged smoke. Volume decreases were not size dependent. Changes in ρ_eff_ with exposure to humidity could be greater than what was measured in this study if smoke were produced with a higher combustion efficiency and had a larger BC/EC component.

### 3.5 Implications, Limitations, and Recommendations

In this study, we measured changes in smoke properties due to oxidative aging and exposure to humidity. During our measurements of chemical composition, we observed SOA formation and increases in OC mass concentrations after the oxidative aging of primary emissions. We also determined that aged smoke takes up more water than fresh smoke at the same humidity levels during our ALWC experiments. The increased ability to take up water after aging is likely due to changes in aerosol structure or surface polarity at low and medium RH. At high RH, we observed no changes in surface-area-normalized ALWC with aging, although κ was higher for aged (0.28 ± 0.03) than for fresh (0.23 ± 0.01) particles. Finally, we measured increases in ρ_eff_ of the core of smoke particles following exposure to RH and subsequent drying and also due to oxidative aging. These increased densities were likely due to a combination of restructuring of aerosol components and filling in of voids by SOA during aging.

Density results from this study are generally supported by the literature, but additional chemical composition analyses and low-voltage electron microscopy imaging would be useful to confirm whether a chemical or structural change—or a combination of both—occurs following exposure to humidity. Assuming the greatest structural changes take place in highly branched soot structures, changes in ρ_eff_ with aging and exposure to humidity would likely be more significant if smoke were produced under higher efficiency combustion conditions than what were used in this study. Other groups have found that particles emitted from low-efficiency combustion are homogeneous and spherical and lack internal voids.^28^ However, it is possible that the OC structures generated during this study had some internal voids that were filled with aging and exposure to humidity. Low-voltage imaging could confirm whether OC experienced structural changes or if restructuring was limited to soot alone.

Results from highly controlled chamber studies such as this one are not fully representative of the chemistry and interactions between materials that occur in the atmosphere or inside homes. However, results from this study illustrate the significant differences in aerosol properties that can occur over time and upon transport between environments under varying environmental conditions. Changes in aerosol composition, size distribution, ALWC, ρ_eff_, and other properties can affect particle transport, surface deposition, and fate in indoor and outdoor environments. These changes can take place while particles travel long distances outdoors or upon transport between outdoor and indoor environments, where they encounter varying environmental conditions. Further, these results may help inform us about particle transport in the human respiratory tract, where temperature rapidly increases and RH nears 100%. Small changes in particle size and morphology due to water uptake can impact the potential for deposition in airways and lungs. Changes in deposition and toxicity due to changes in aerosol properties with aging and exposure to RH can have a significant impact on health impacts of exposure, as shown in previous studies.^5,6^ Further research into how health and climate may be affected by changes in aerosol properties is needed.

## Supporting Information Available

Size distribution heat maps of representative fresh and aged smoke, GC-MS chromatograms of fresh and aged smoke, and values of ALWC, volume growth factor, κ, and ρ_eff_ for all experimental conditions (PDF)
