# Supplementary material for: Impacts of Aging and Relative Humidity on Properties of Biomass Burning Smoke Particles
Source: ACS EST Air. 2024 Dec 6;2(1):109–18. doi: 10.1021/acsestair.4c00224 (PMC11730893; doi:10.1021/acsestair.4c00224)
Supplement: Supplementary file 1 — ea4c00224_si_001.pdf [file ea4c00224_si_001.pdf]

Supplemental Information for

Impacts of Aging and Relative Humidity on Properties of Biomass Burning Smoke Particles

Sofie K. Schwink[1], Liora E. Mael[2◊], Thomas H. Dunnington[3], Maximilian J. Schmid[1], Jonathan M. Silberstein[2], Andrew Heck[2], Nicholas Gotlib[2], Michael P. Hannigan[1,2], Marina E. Vance[1,2]*

[1]Environmental Engineering Program, University of Colorado Boulder, 1111 Engineering Drive, Boulder, CO 80309-0428, USA.

[2]Department of Mechanical Engineering, University of Colorado Boulder, 1111 Engineering Drive, Boulder, CO 80309-0427, USA.

[3]Department of Aerospace Engineering, University of Colorado Boulder, 429 UCB, 3775 Discovery Drive, Boulder, CO 80303, USA.

*Corresponding Author, marina.vance@colorado.edu

[◊]Now at: Winterthur Museum, Garden and Library, University of Delaware, Newark, DE, USA

List of figures

Figure S1. Size distribution heat map from a representative fresh smoke experiment at low RH.
Figure S2. Size distribution heat map from a representative aged smoke experiment at low RH.
Figure S3. Chromatogram from fresh and aged smoke experiments normalized by OC concentration. Two filters for each condition were combined for this analysis.
Figure S4. Representative particle size distributions for fresh and aged smoke at low RH from ALWC experiments. The larger size distribution for each condition is from the wet SMPS and the smaller size distribution is from the dry SMPS. A correction has been applied to data from the wet SMPS.
Figure S5. Volume growth factors from ALWC experiments for all experimental conditions.
Figure S6. Kappa values from ALWC experiments for all experimental conditions.
Figure S7. Size-resolved effective density for all experimental conditions, including medium RH. The top panel shows results for fresh smoke and the bottom panel for aged smoke.

List of tables

Table S1. Average peaks in size distribution for all experiments.
Table S2. Average ALWC as a fractional portion of total aerosol volume.
Table S3. Effective density (ρ_{eff}, g cm^{-3}) of smoke aerosols with associated water across all size bins at low and high RH. No effective density distributions were observed at the 340 nm particle size. These experiments were performed once and not performed at medium RH.
Table S4. Effective density (ρ_{eff}, g cm^{-3}) of dried smoke aerosols under all experimental conditions. No effective density distributions were observed at the 340 nm particle size. Averages of two experiments shown ± standard deviation.

Table S1. Average peaks in size distribution for composition experiments, ALWC experiments with a dryer, ALWC without a dryer, and density experiments. Average diameters shown ± standard deviation.

Condition	Experiment			
	Composition experiments D (nm)	ALWC with dryer D (nm)	ALWC without dryer D (nm)	ρ_{eff} D (nm)
**Fresh smoke, low RH**	140 ± 21	149 ± 4	151 ± 0	153 ± 31
**Aged smoke, low RH**	150 ± 28	160 ± 12	169 ± 9	158 ± 24
**Fresh smoke, medium RH**	-	154 ± 4	163 ± 8	139 ± 18
**Aged smoke, medium RH**	-	172 ± 5	181 ± 9	163 ± 8
**Fresh smoke, high RH**	-	123 ± 25	132 ± 27	140 ± 32
**Aged smoke, high RH**	-	151 ± 5	166 ± 4	151 ± 0

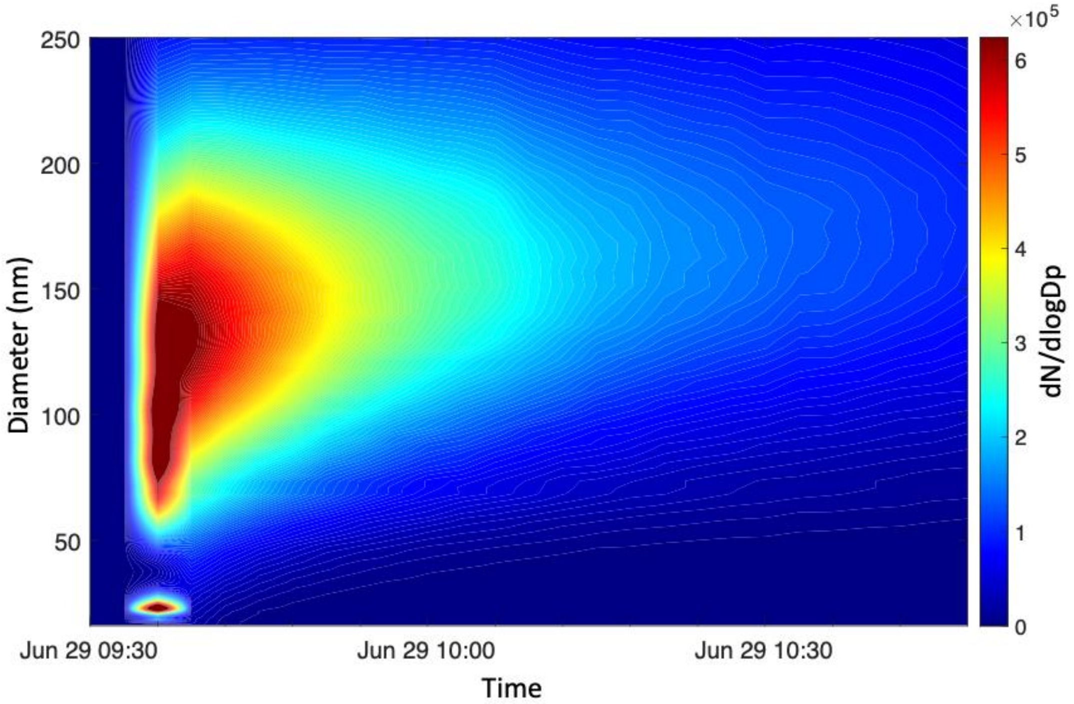

Figure S1. Size distribution heat map from a representative fresh smoke experiment at low RH.

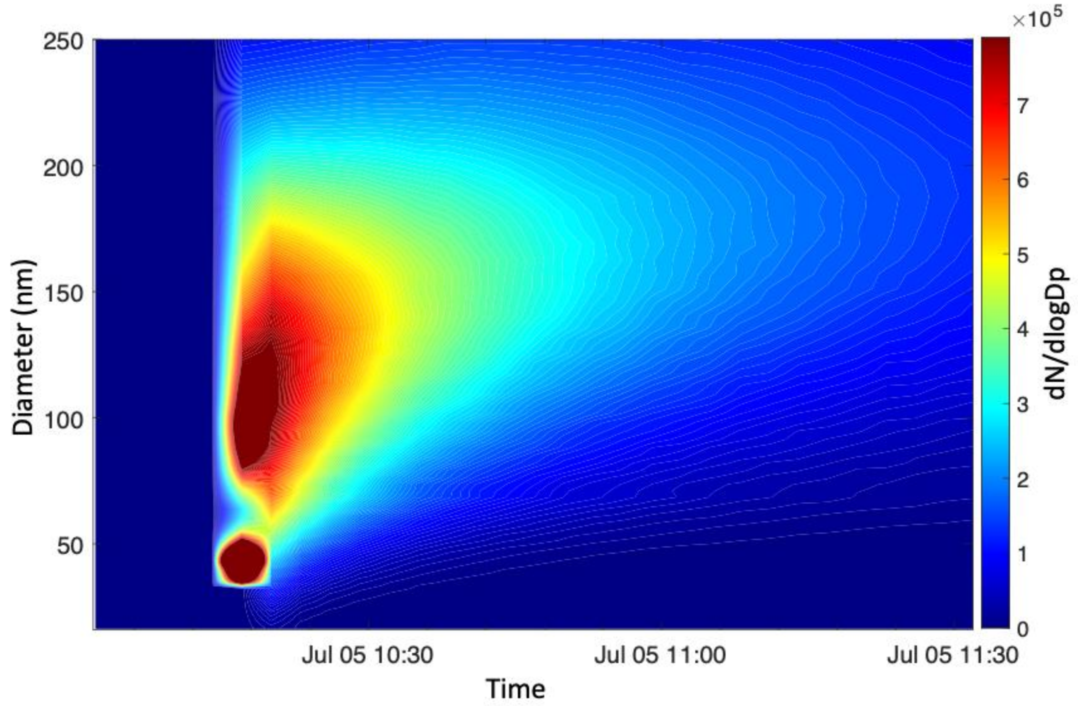

Figure S2. Size distribution heat map from a representative aged smoke experiment at low RH.

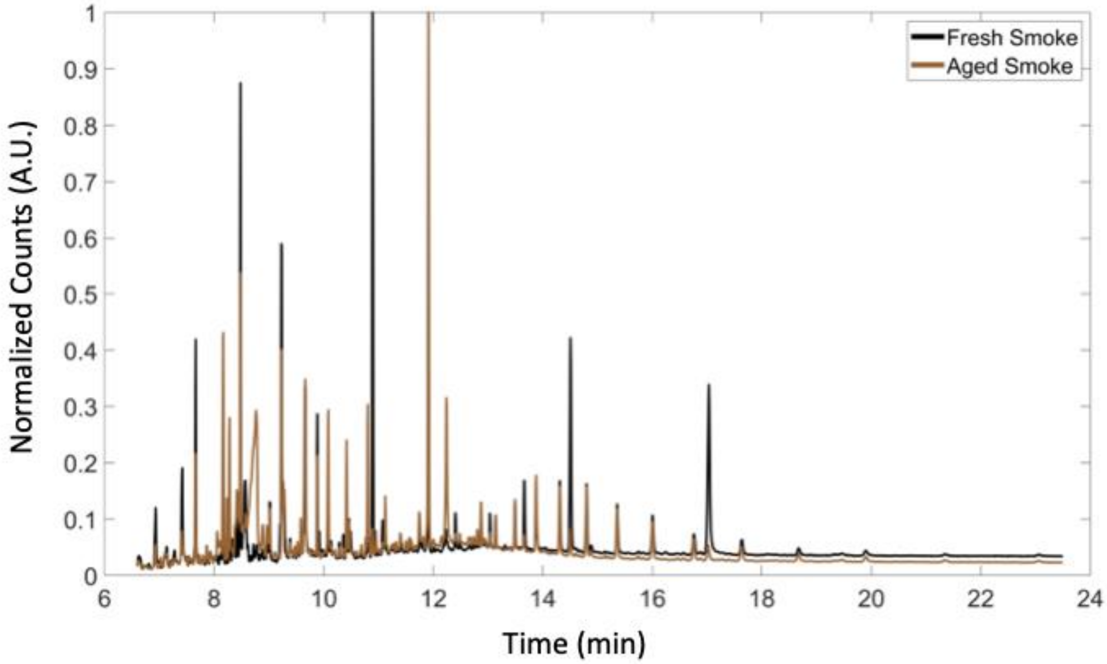

Figure S3. Chromatogram from fresh and aged smoke experiments normalized by OC concentration. Two filters for each condition were combined for this analysis.

Table S2. Average ALWC as a fractional portion of total aerosol volume. Averages shown ± standard deviation.

Experiment	ALWC (volume fraction)
**Fresh smoke, low RH**	0.05 ± 0.08
**Aged smoke, low RH**	0.13 ± 0.01
**Fresh smoke, medium RH**	0.19 ± 0.02
**Aged smoke, medium RH**	0.21 ± 0.05
**Fresh smoke, high RH**	0.21 ± 0.06
**Aged smoke, high RH**	0.30 ± 0.09

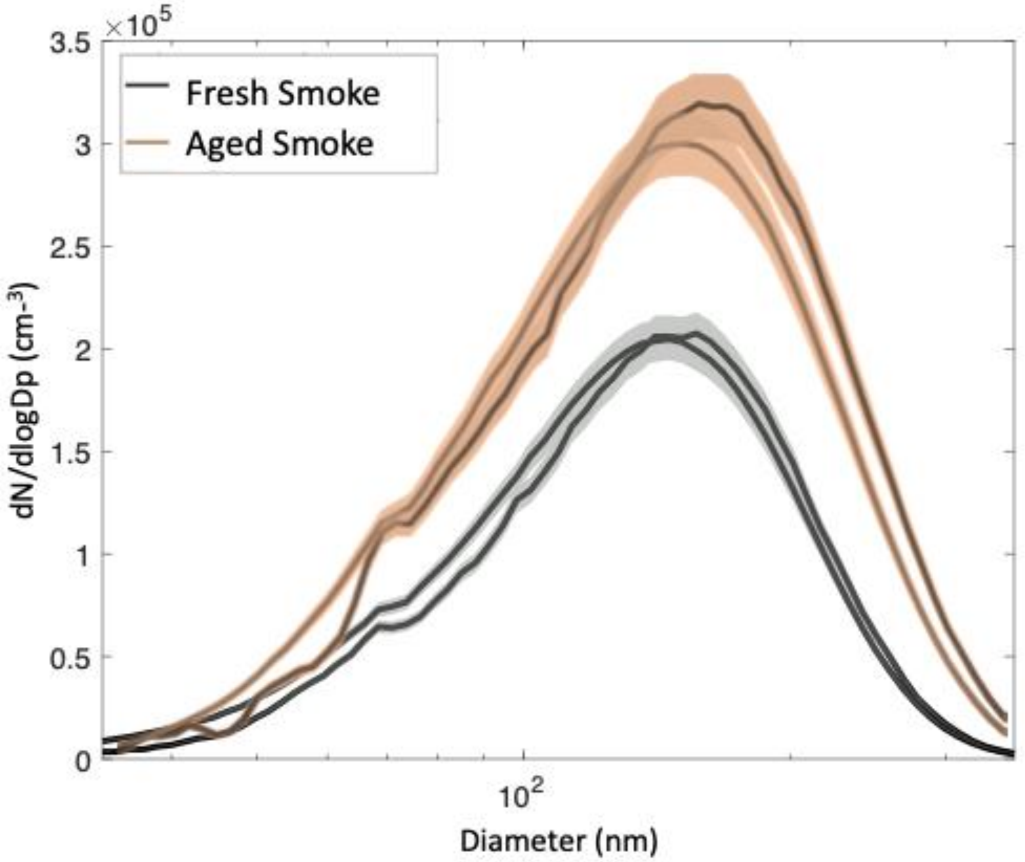

Figure S4. Average particle size distributions for fresh and aged smoke at low RH from ALWC experiments. Solid lines represent the average of 75 minutes of experiments (~25 size distributions) and the shaded regions represent a ± 5% error region. The larger size distribution for each condition is from the wet SMPS and the smaller size distribution is from the dry SMPS. A correction has been applied to data from the wet SMPS.

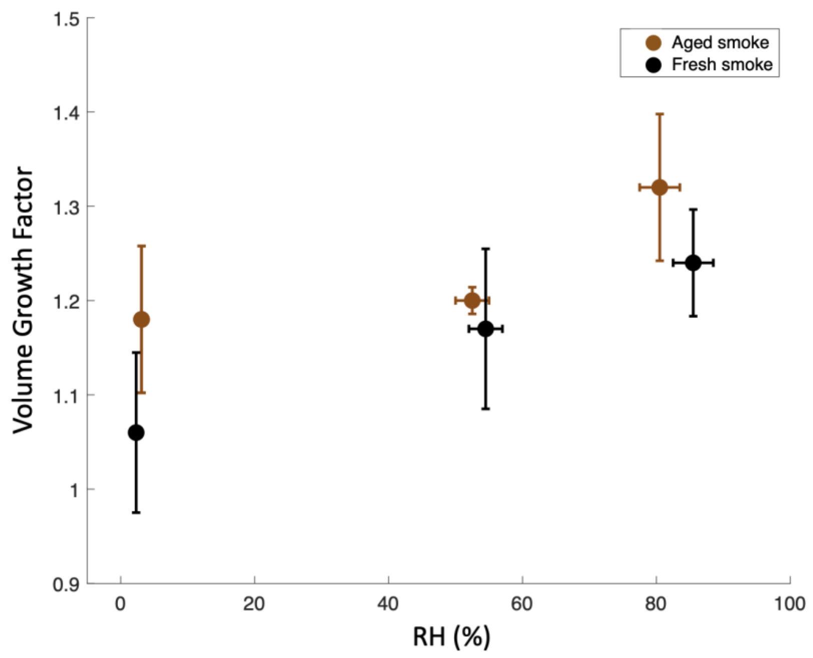

Figure S5. Volume growth factors from ALWC experiments for all experimental conditions. Averages shown ± standard deviation.

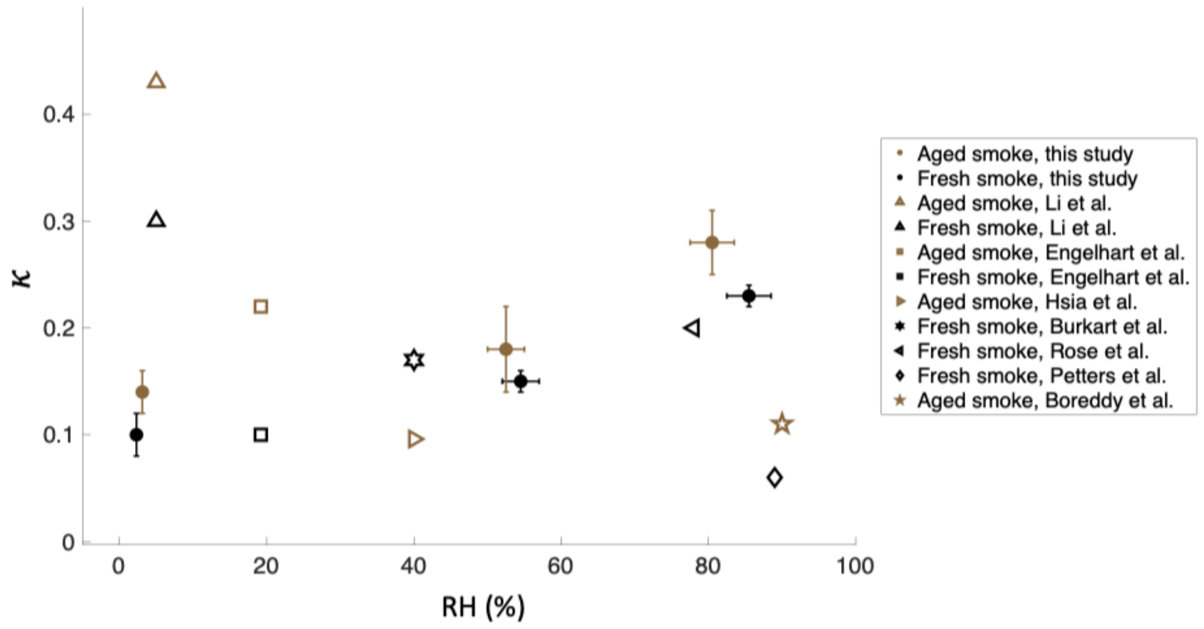

Figure S6. Kappa (κ) values from all ALWC experiments. Averages of two experiments shown ± standard deviation.

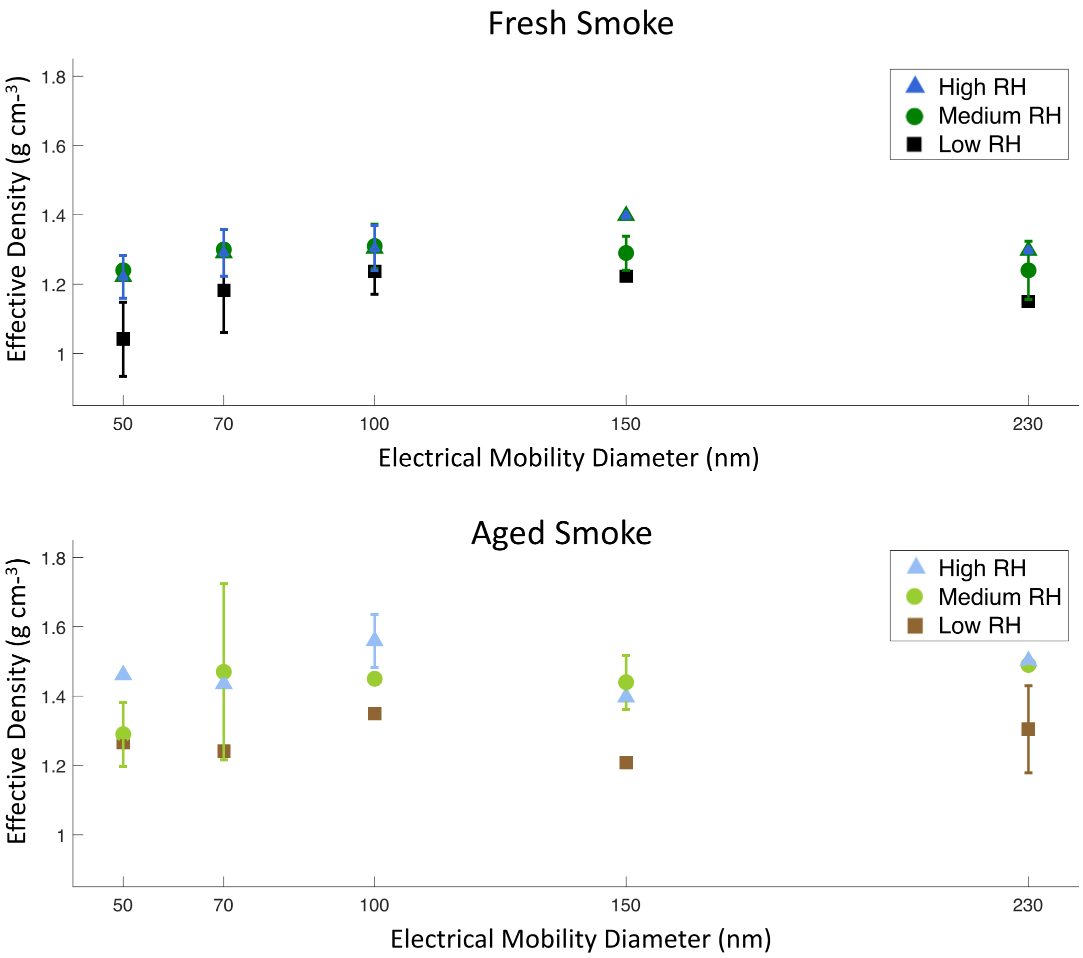

Figure S7. Size-resolved effective density for all experimental conditions, including medium RH. The top panel shows results for fresh smoke and the bottom panel for aged smoke. Averages shown ± standard deviation.

Table S3. Effective density (ρ_{eff}, g cm^{-3}) of smoke aerosols with associated water across all size bins at low and high RH. No effective density distributions were observed at the 340 nm particle size. These experiments were performed once and not performed at medium RH.

Experiment	Particle size				
	50 nm	70 nm	100 nm	150 nm	230 nm
Fresh smoke, low RH	1.05	1.21	1.32	1.17	0.94
Aged smoke, low RH	1.09	1.07	1.27	1.21	1.12
Fresh smoke, high RH	1.08	1.21	1.32	1.17	1.09
Aged smoke, high RH	1.27	1.24	1.37	1.21	1.12

Table S4. Effective density (ρ_{eff}, g cm^{-3}) of dried smoke aerosols under all experimental conditions. No effective density distributions were observed at the 340 nm particle size. Averages of two experiments shown ± standard deviation.

Experiment	Particle size				
	50 nm	70 nm	100 nm	150 nm	230 nm
Fresh smoke, low RH	1.04 ± 0.11	1.18 ± 0.12	1.24 ± 0.07	1.22 ± 0.00	1.15 ± 0.00
Fresh smoke, medium RH	1.24 ± 0.00	1.30 ± 0.01	1.31 ± 0.06	1.29 ± 0.05	1.24 ± 0.09
Fresh smoke, high RH	1.22 ± 0.06	1.29 ± 0.07	1.30 ± 0.07	1.40 ± 0.00	1.30 ± 0.00
Aged smoke, low RH	1.27 ± 0.00	1.24 ± 0.00	1.35 ± 0.00	1.21 ± 0.00	1.30 ± 0.13
Aged smoke, medium RH	1.29 ± 0.00	1.47 ± 0.01	1.45 ± 0.00	1.44 ± 0.08	1.49 ± 0.01
Aged smoke, high RH	1.46 ± 0.00	1.44 ± 0.00	1.56 ± 0.08	1.40 ± 0.00	1.50 ± 0.00
